# Potential for substitution of mental health care towards family practices: an observational study

**DOI:** 10.1186/s12875-017-0586-4

**Published:** 2017-01-31

**Authors:** Tessa Magnée, Derek P. de Beurs, Richard Boxem, Dinny H. de Bakker, Peter F. Verhaak

**Affiliations:** 1 Netherlands Institute of Health Services Research (NIVEL), PO Box 1568, 3500 BN Utrecht, The Netherlands; 2 The Dutch Healthcare Authority, Utrecht, The Netherlands; 3 Tilburg University, Scientific Centre for Transformation in Care and Welfare (TRANZO), Tilburg, The Netherlands; 4 Department of General Practice, Groningen University, Groningen, The Netherlands

**Keywords:** Family practice, Mental health, Psychiatric disorders, Primary care, Health services

## Abstract

**Background:**

Substitution is the shift of care from specialized health care to less expensive and more accessible primary health care. It seems promising for restraining rising mental health care costs. The goal of this study was to investigate a potential for substitution of patients with psychological or social problems, but without severe psychiatric disorders, from Dutch specialized mental health care to primary care, especially family practices.

**Methods:**

We extracted anonymized data from two national databases representing primary and specialized care in 2012. We calculated the number of patients with and without psychiatric disorder per 1,000 citizens in three major settings: family practices, primary care psychologists, and specialized care. Family physicians recorded psychopathology using the International Classification of Primary Care, while psychologists and specialists used the Diagnostic and Statistical Manual of Mental Disorders, 4th Edition.

**Results:**

Considerable numbers of patients without a diagnosed DSM-IV psychiatric disorder were treated by primary care psychologists (32.8%) or in specialized care (20.8%). Over half of the patients referred by family physicians to mental health care did not have a psychiatric disorder.

**Conclusion:**

A recent reform of Dutch mental health care, including new referral criteria, will likely increase the number of patients with psychological or social problems that family physicians have to treat or support. Enabling and improving diagnostic assessment and treatment in family practices seems essential for substitution of mental health care.

**Electronic supplementary material:**

The online version of this article (doi:10.1186/s12875-017-0586-4) contains supplementary material, which is available to authorized users.

## Background

Substitution is the shift of care from specialized health care to less expensive and more accessible (primary) health care. This method seems promising for restraining rising health care costs [1]. The urgency for substitution in Dutch mental health care is high, as costs have increased significantly in the last years in the Netherlands [2]. Presumably, some patients treated in specialized mental health care do not have a severe psychiatric disorder, do not genuinely need treatment from specialists, and could be treated in a primary care setting instead. Previous studies suggest that up to one third or even one half of the patients who receive treatment in mental health care do not meet the formal criteria for a psychiatric disorder [3–5], although they may have other need indicators which justify treatment.

The WHO underlines the importance of integrating mental health care into general health care settings [6]. However, the consequences of enhanced primary mental health care for health care utilization and costs remain unclear. Previous reviews on consultation of mental health professionals by primary care professionals suggest that it is as effective as care as usual in improving clinical outcomes [7], but that it may also reduce utilization of health care services [8]. A Cochrane review on counselling provided in primary care in the UK [9] concluded that patients were satisfied, and that counselling was associated with enhanced clinical effectiveness compared to care as usual (but only in short-term). However, counselling in primary care did not seem to reduce overall healthcare costs. A Cochrane review on mental health workers integrated in primary care concluded that their presence might decrease consultation rates of other primary care professionals, prescriptions of psychotropic drugs, and referrals to specialists [10]. However, effects were modest and results were not consistent amongst all included studies. Moreover, economic significance of the results remained unclear. Some studies in the UK and the US suggest that enhanced primary mental health care is cost-effective [11, 12], but also that it requires (extra) direct financial investments over the short-term [13].

**Fig. 1:**
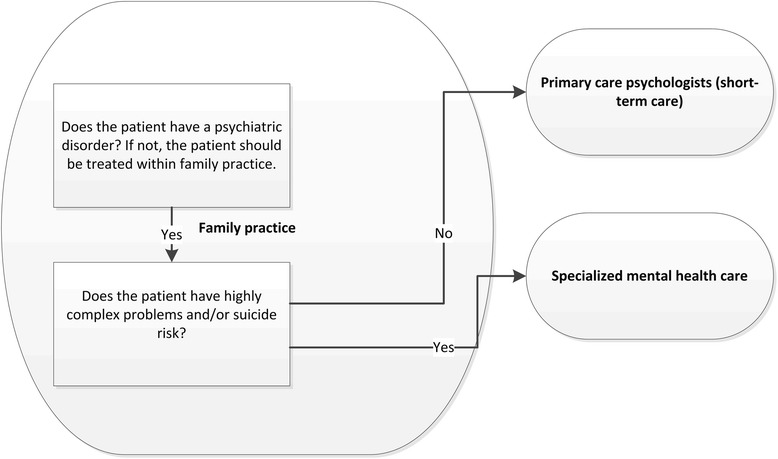
Mental health care in the Netherlands: referral rules. Shows new referral rules for Dutch family physicians regarding patients with mental health problems

In 2014, the Dutch government introduced a reform of mental health care to promote the substitution of mental health care towards general health care settings, especially towards family practices. According to new referral criteria (Fig. 1), all patients with only mild psychological symptoms or social problems should be treated within family practices. Family physicians (FPs) are no longer allowed to refer patients without an actual psychiatric disorder to mental health care, consisting of primary care psychologists (PCPs) and specialized mental health care. Patients with a psychiatric disorder and non-complex problems (no comorbidity with for example a personality disorder or complicating psychosocial problems) should be referred to primary care psychologists (since the reform labeled as ‘basic mental health care’) for short-term care, while patients with complex problems should be referred to specialized mental health care for (longer term) treatment by a multidisciplinary team.

To prepare FPs for the treatment of more patients with mental health problems, the function of the mental health nurse (MHN) was introduced. Increasing numbers of Dutch FPs collaborate with an MHN: a nurse with mental health expertise, or a psychologist. MHNs can perform diagnostic research and provide short-term care [14], but they may also indirectly improve FPs’ knowledge and skills in the field of mental health. Short-term psychological interventions provided in the primary care setting are accessible, seem effective, and lead to high patient satisfaction [9, 15–19]. Examples of such interventions are self-help programs, counseling, problem solving therapy, and brief cognitive behavior therapy.

Although many previous studies have evaluated the integration of mental health care into primary care, it is not clear what effects a national reform aimed at a shift of patients from specialized care towards primary care might have. The aim of this study was to investigate the potential for substitution of Dutch mental health care in the context of the new referral rules for FPs. Our central research question was: what is the potential for substitution of mental health care from specialized care and PCPs to family practices, and from specialized care to PCPs?

We investigated: (1) how many patients with and without psychiatric disorders were seen in the three mental health care settings before the reform, (2) how many patients with and without psychiatric disorders were referred by FPs and PCPs to primary and specialized care before the reform, and (3) how many patients with and without complex problems were treated by PCPs and in specialized care before the reform.

## Methods

### Data sources

In an observational, cross-sectional study, we extracted anonymized patient data from two national databases, to describe primary care, including family practices and primary care psychologists, and specialized mental health care in 2012.

Data on primary care were extracted from electronic health records of caregivers participating in the NIVEL Primary Care Database (NIVEL-PCD). All caregivers participating in the NIVEL-PCD, including FPs and PCPs, routinely record care they deliver to patients. Family practices participating in the NIVEL-PCD are representative of Dutch family practices [20], although group practices and practices in non-urban areas are somewhat overrepresented compared to national numbers [21]. The patient populations of the family practices are representative of the Dutch population according to sex and age. Only data from practices with the most complete records were used for this study (*n* = 180 practices; *n* = 685,337 patients). Not all FPs keep complete records of referrals. A smaller number of practices were included in the analyses on referrals (*n* = 25 practices; *n* = 90,734 patients). Practices with complete referral records did not differ from other practices regarding practice type, degree of urbanization, or practice size.

In 2012, 543 primary care psychologists were participating in the NIVEL PCD, providing care to 45,947 patients. The database covered 14.6% of all patients treated by primary care psychologists working in the Netherlands [22].

Data on specialized care were extracted from a national database for specialized care [23]. This database covers all caregivers, mostly psychiatrists and psychologists, working in Dutch specialized mental health care institutions, as well as solo operating entrepreneurs. Professionals working in specialized care are obliged by Dutch law to record all provided care that is paid for by health insurers in the national database. Therefore, virtually all Dutch patients treated in specialized mental health care were represented in this database.

### Patient data

We extracted data on the number of seen or treated and referred patients and their diagnoses. To facilitate comparability between the three settings, all extracted numbers were converted to numbers of patients per 1,000 citizens based on the Dutch population number of 2012 [24].

FPs use the International Classification of Primary Care (ICPC) system to record diagnoses of patients within chapters of diseases during consultations. Within each ICPC chapter, a subdivision is made between symptoms (codes 01–29) and diseases or disorders (codes 70–99), although the Z chapter (social problems) is limited to symptom codes. Only patients with at least one consultation at the family practice with a diagnosis concerning psychological problems (P chapter) or social problems (Z chapter) were included in the study. We distinguished between patients with a psychiatric disorder (P70–P99) and patients without a psychiatric disorder (psychological symptoms, P01–P29, or social problems, Z01–Z29).

PCPs and caregivers in specialized care record a DSM-IV diagnosis for each patient during treatment. Diagnostic assessment usually takes place during an intake phase, when a wide range of diagnostic instruments may be used. The DSM-IV is a globally used classification system for psychiatric disorders, covering five axes [25]. Axis 1 represents the primary disorder or psychopathology of the patient. Axes 2 to 4 represent comorbid, underlying, or related problems. Caregivers use axis 2 to report personality disorders, axis 3 for somatic diseases, axis 4 for psychosocial problems, and axis 5 for the level of (dis)functioning of the patient. The latter was not included in this study, as it was not available for all patients in specialized care. We used the axis 1 diagnosis to determine if patients of PCPs or in specialized care had a psychiatric disorder or not. Patients had problems of higher complexity if they had comorbid problems on axis 2 (a personality disorder), axis 3 (somatic problems), or axis 4 (psychosocial problems).

## Results

### Patients with and without disorders in three settings

**Fig. 2:**
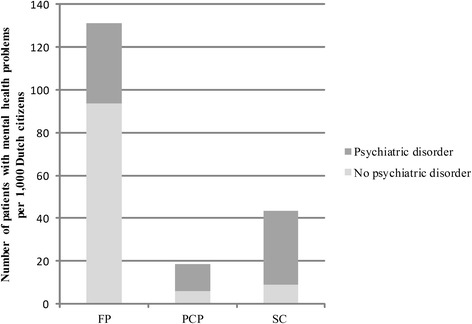
Number of patients with and without a psychiatric disorder in three settings per 1,000 Dutch citizens in 2012. Shows how many patients with mental health problems were treated in 2012 by family physicians, primary care psychologists, and in specialized care in the Netherlands. Notes: FP = family practice, PCP = primary care psychologist, SC = specialized care

Figure 2 shows the number of patients with and without psychiatric disorders in each of the three major settings of Dutch mental health care. FPs saw 131.0 patients with psychological problems per 1,000 citizens, mostly patients without psychiatric disorders (71.3%). FP patients often had psychological symptoms such as anxious feelings, or social problems such as problems with their partner (Additional file 1: Table S1).

PCPs and caregivers in specialized care treated a smaller number of patients compared to FPs, 18.7 and 43.7 patients per 1,000 citizens, respectively. A considerable number of patients treated by primary care psychologists or in specialized care did not have a psychiatric disorder, 32.8% and 20.8%, respectively. In total, 15.3 patients without a disorder per 1,000 citizens were treated by primary care psychologists or in specialized care. These patients were for example diagnosed with other worries or problems, adjustment problems, or had no diagnosis (Additional file 2: Table S2 and Additional file 3: Table S3).

### Referrals

**Fig. 3:**
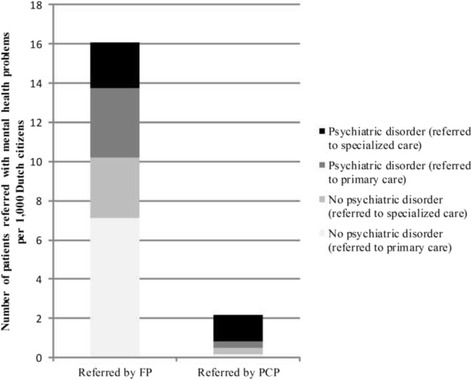
Number of patients referred with and without psychiatric disorder per 1,000 Dutch citizens in 2012. Shows how many patients with mental health problems were referred in 2012 by family physicians and by primary care psychologists to primary and specialized care. Notes: FP = family physician. PCP = primary care psychologist. Primary care: (other) FP, (other) PCP, or social work

Figure 3 shows how many patients with and without psychiatric disorders were referred by FPs and PCPs to primary and specialized care (Additional file 4: Table S4 shows exact numbers). FPs in total referred 16.1 patients with psychological and social problems per 1,000 citizens to primary or specialized care, which is 12.3% of all the FP patients with psychological and social problems. Over half of the referred FP patients (63.2%) did not have a psychiatric disorder. PCPs in total referred 2.2 patients per 1,000 citizens to primary and specialized care, which is 11.7% of all patients they treated. Of the referred PCP patients, around one fifth (22.4%) did not have a psychiatric disorder.

### Complexity of problems

**Fig. 4:**
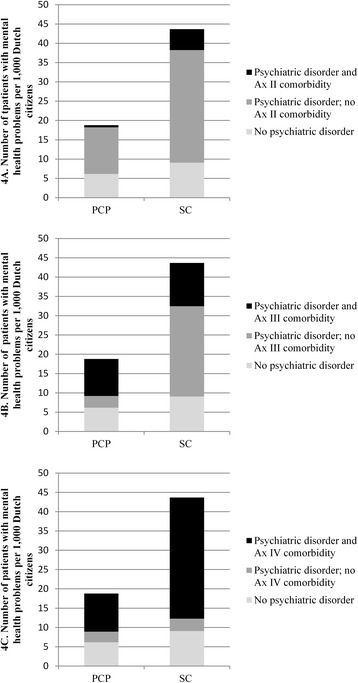
Number of patients with and without comorbidity in two settings per 1,000 Dutch citizens in 2012. Shows how many patients with mental health problems treated in 2012 by primary care psychologists or in specialized care had comorbid problems. Notes: PCP = primary care psychologist. SC = specialized care

Figure 4 shows that many patients treated by PCPs had comorbidity, mostly a psychiatric disorder combined with psychosocial problems (78.3%), or somatic problems (75.9%). Only one in every twenty PCP patients had a combination of a psychiatric disorder and a personality disorder (4.5%). Most patients treated in specialized care had comorbidity as well; most patients with a disorder also had psychosocial problems (90.8%). Fewer patients treated in specialized care had a psychiatric disorder combined with somatic problems (32.4%) or with a personality disorder (15.7%).

## Discussion

### Summary of findings

Prior to the recent reform of Dutch mental health care, a significant number of patients treated by PCPs (approximately one third) and in specialized care (approximately one fifth) did not have a diagnosed DSM-IV disorder. Over half of the patients with psychological and social problems referred by FPs to mental health professionals did not have a diagnosis of a psychiatric disorder. Most patients with a disorder in specialized care had complex problems, expressed by comorbidity on axis 4 of the DSM-IV (psychosocial problems). Comorbidity on axis 3 (somatic problems) or axis 2 (personality disorders) was less common.

### Potential for substitution from PCPs and specialized care to family practice

The proportion of patients without a psychiatric disorder in mental health care observed in this study is in line with previous research [3–5]. If we evaluate this study as a baseline measure of the reform of the Dutch mental health care system, we expect that a part of the patients previously referred to and treated in mental health care from now on will receive treatment within family practices. Nurses with mental health expertise, who work in increasing numbers of family practices in the Netherlands, may be of crucial importance in the diagnostic assessment and treatment of those patients.

An apparent discrepancy was observed between the number of patients without a psychiatric disorder who were referred by FPs (over 60%) and the lower number of patients without a psychiatric disorder who were treated by PCPs and in specialized care (20.8% and 32.8%). It is highly plausible that (final) diagnostic assessment often takes place *after* referral, We have to consider the possibility that some of the patients might be falsely diagnosed after referral. Although the DSM-IV is highly useful, its limitations are widely debated, and it might stimulate overdiagnosis [26, 27]. Moreover, the Dutch health insurance only covers PCP and specialist treatment for patients with a DSM-IV disorder, which may encourage overdiagnosis even further. Remarkably, FPs now also face the risk of overdiagnosis, as the new referral criteria make a psychiatric disorder a necessity for every referral to mental health care.

Some of the patients recently treated by mental health care professionals or referred by GPs may not have had a diagnosed psychiatric disorder, but they may certainly have been in need of treatment. Previous research showed that approximately half of the patients in mental health care had no psychiatric disorder, but showed other important indicators of need for treatment, such as multiple subthreshold disorders, a recent stressor, psychosocial problems, or suicidal behavior [3, 4]. If we assume this is also true for the patients included in this study, the new referral criteria for FPs regarding psychiatric disorders could have an unwanted effect. Some patients, who are certainly in need of mental health care, but who do not meet formal criteria for a psychiatric disorder, may be deprived of appropriate treatment.

In either way, enabling and improving diagnostic assessment in family practices seems essential for substitution of mental health care. Good quality diagnostic assessment in family practice could facilitate the correct adoption of the new referral criteria. It could also improve the continuity of care from the patient’s perspective. A screening instrument may be helpful, for example a symptom severity assessment [28]. MHNs could improve diagnostic assessment, both by performing it themselves or by indirectly improving FPs’ knowledge and skills through collaboration.

### Potential for substitution from specialized care to PCPs

We expect substitution towards PCPs to a lesser extent compared to substitution toward family practices, as most patients treated in specialized care with a disorder had at least comorbid psychosocial problems. However, it is debatable to what extent psychosocial problems genuinely represent a complicating factor, as they are often temporary and can be solved in a relatively straightforward way. Other comorbid diagnoses, such as personality disorders, were observed less frequently, but they are more likely to complicate treatment [29]. From this perspective, more potential for substitution from specialized care toward PCPs may exist than observed in this study.

### Health care costs

Restraining costs was one the most important reasons for the recent Dutch mental health care reform. Our study indicates that, in the near future, at least some of the patients with mental health problems may be treated in (less expensive) primary care instead of in specialized care. This may result in a cost reduction. However, the recent Dutch reform might also have unintended cost effects. The accessibility of mental health care is likely to be improved, and some patients that might not have been treated at all before may now receive treatment in family practice. Moreover, it is unknown how many of the patients initially treated in family practice afterwards still need a referral to specialized care. Future research following patients through the different echelons of mental health care is needed to evaluate cost effects.

### International relevance

The WHO states it is important to redirect funding towards community-based services, including the integration of mental health care into general health care settings [6]. Various health care system characteristics influence the role of the FP in mental health care [30, 31], for example the referral system, FP workload and mental health expertise, financial regulations, and patient expectations. These factors vary strongly between countries, and influence a possible shift of mental health care from specialized care to primary care. Potential for substitution is likely to exist in other countries besides the Netherlands, as was shown by the numbers of patients without a psychiatric disorder treated in mental health care [3, 4], and by the numerous international studies evaluating the integration of mental health care into primary care [7–12]. FPs in the UK [32] and Canada [33] are collaborating with professionals similar to mental health nurses, which might enable substitution.

### Strengths and limitations

As this is a descriptive study, we cannot draw any conclusions on causality. A major strength of this study is that we were able to combine two national databases, thereby including a very large number of patients in primary care and virtually all patients in specialized care. This study can function as a baseline measure for the recent Dutch mental health care reform.

Caregivers working in different settings vary in their skills to recognize and diagnose mental health problems. Caregivers working in different settings use different classification systems (ICPC and DSM-IV), which may complicate comparability between the settings. Previous research has shown that GPs do not always recognize psychological problems, or that they may be aware of mental health problems but do not label patients with a specific psychological diagnosis [34]. Only about half of all persons with mental disorders had contact with their GP in the last six months [35]. The diagnoses of persons who do not seek help are not coded and were thus not included in this study.

Professionals working in mental health care sometimes postpone giving a diagnosis. This could mean some of the patients included in our study may have been diagnosed later on. However, data were extracted a considerable amount of time after treatment (2014 vs. 2012). We categorized patients without a diagnosis as patients without a disorder, as we assume that the complaints of many of these patients were not severe enough to have led to an official diagnosis of a disorder within a decent amount of time. PCPs had the possibility of explicitly choosing between “no axis 1 disorder” or “diagnosis postponed”. The latter was only used for a one out of five patients without a diagnosis (Additional file 2: Table S2).

We were unfortunately not able to, besides the DSM-IV axis 2 to axis 4 comorbidity, include any other complicating factors, such as suicide risk. These factors were not routinely recorded by caregivers in any setting.

## Conclusions

A recent reform of Dutch mental health care, including new FP referral criteria, will likely lead to a considerable increase in patients with psychological or social problems that have to be treated within family practices. Enabling and improving diagnostic assessment and treatment in family practices seems essential for substitution of mental health care.

## Additional files

Additional file 1:
**Table S1.** Number of patients seen for psychological or social problems at Dutch family practices per 1,000 citizens in 2012. Contains a table with the number of patients with mental health problems treated in 2012 in family practices, according to diagnosis. (DOCX 14 kb)
Additional file 2:
**Table S2.** Number of patients treated by primary care psychologists per 1,000 Dutch citizens in 2012. Contains a table with the number of patients with mental health problems treated in 2012 by primary care psychologists, according to diagnosis. (DOCX 16 kb)
Additional file 3:
**Table S3.** Number of patients treated in secondary care per 1,000 Dutch citizens in 2012. Contains a table with the number of patients with mental health problems treated in 2012 in specialized care, according to diagnosis. (DOCX 16 kb)
Additional file 4:
**Table S4.** Number of patients referred by FPs and PCPs per1,000 Dutch citizens in 2012. Contains a table with the number of patients with mental health problems referred in 2012 by family physicians and by primary care psychologists to primary and specialized care. (DOCX 16 kb)

## Abbreviations

FP: Family physician
ICPC: International Classification of Primary Care
MHN: Mental health nurse
NIVEL: Netherlands Institute for Health Services Research
NIVEL PCD: NIVEL Primary Care Database
PCP: Primary care psychologist
SC: Specialized care
