# Supplementary material for: Potential for substitution of mental health care towards family practices: an observational study
Source: BMC Fam Pract. 2017 Jan 31;18:10. doi: 10.1186/s12875-017-0586-4 (PMC5282718; doi:10.1186/s12875-017-0586-4)
Supplement: Additional file 1: — Table S1. Number of patients seen for psychological or social problems at Dutch family practices per 1,000 citizens in 2012. Contains a table with the number of patients with mental health problems treated in 2012 in family practices, according to diagnosis. (DOCX 14 kb) [file 12875_2017_586_MOESM1_ESM.docx]

Additional file 1: Table S1. Number of patients seen for psychological or social problems at Dutch family practices per 1,000 citizens in 2012

| Anxious feelings (1801) | 9.16 |
| --- | --- |
| Stress (1802) | 5.11 |
| Depressive feelings (1803) | 5.85 |
| Sleeping problems (1806) | 13.57 |
| Alcohol misuse (1815) | 2.08 |
| Tobacco misuse (1817) | 5.47 |
| Concentration/memory problems 1820) | 3.83 |
| Hyperactive child (1821) | 3.78 |
| Other worries child behavior (1822) | 3.96 |
| Learning problem (1824) | 2.68 |
| Other psychological symptoms | 11.77 |
| Work problems (2605) | 2.46 |
| Relational problem partner (2612) | 4.92 |
| Problem ill partner (2614) | 2.07 |
| Loss of partner (2615) | 3.03 |
| Other social problems | 13.70 |
| **No psychiatric disorder (total)** | **93.44** |
| Dementia/Alzheimer (1870) | 2.41 |
| Anxiety disorder (1874) | 7.12 |
| Depression (1876) | 12.41 |
| Neurasthenia (1878) | 6.35 |
| Personality disorder (1880) | 1.43 |
| Other psychiatric disorders | 7.87 |
| **Psychiatric disorder (total)** | **37.59** |
| **Total** | **131.03** |

Notes: other psychological symptoms are all ICPC codes between P01 and P29 that are not shown in the table. Other social problems are all ICPC codes between Z01 and Z29 that are not shown in the table. Other psychiatric disorders are all ICPC codes between P70 and P99 that are not shown in the table.
