# Supplementary material for: Potential for substitution of mental health care towards family practices: an observational study
Source: BMC Fam Pract. 2017 Jan 31;18:10. doi: 10.1186/s12875-017-0586-4 (PMC5282718; doi:10.1186/s12875-017-0586-4)
Supplement: Additional file 2: — Table S2. Number of patients treated by primary care psychologists per 1,000 Dutch citizens in 2012. Contains a table with the number of patients with mental health problems treated in 2012 by primary care psychologists, according to diagnosis. (DOCX 16 kb) [file 12875_2017_586_MOESM2_ESM.docx]

Additional file 2: Table S2. Number of patients treated by primary care psychologists per 1,000 Dutch citizens in 2012

|  | **Total** | Axis 2 | | | | Axis 3 | | | Axis 4 | |
| --- | --- | --- | --- | --- | --- | --- | --- | --- | --- | --- |
| Axis 1 |  | | Comorbidity | No comorbidity | Comorbidity | | No comorbidity | Comorbidity | | No comorbidity |
| No disorder | 2.40 | | 0.06 | 2.34 | 0.31 | | 2.08 | 1.83 | | 0.57 |
| No diagnosis yet | 0.68 | | 0.01 | 0.67 | 0.11 | | 0.56 | 0.54 | | 0.13 |
| Adjustment problems | 0.92 | | 0.03 | 0.90 | 0.67 | | 0.71 | 0.78 | | 0.14 |
| Other worries or problems | 2.17 | | 0.05 | 2.11 | 0.35 | | 1.82 | 1.85 | | 0.31 |
| **No DSM-IV Axis 1 disorder (total)** | **6.17** | | **0.15** | **6.02** | **0.99** | | **5.18** | **5.01** | | **1.16** |
| Mood disorder | 5.70 | | 0.24 | 5.46 | 1.28 | | 4.43 | 4.76 | | 0.94 |
| Anxiety disorder | 4.21 | | 0.19 | 4.02 | 0.92 | | 3.29 | 2.96 | | 1.25 |
| Substance or alcohol related disorder | 0.13 | | 0.02 | 0.11 | 0.02 | | 0.10 | 0.11 | | 0.01 |
| Dementia | 0.03 | | 0.00 | 0.02 | 0.02 | | 0.01 | 0.02 | | 0.01 |
| Somatoform disorder | 1.03 | | 0.03 | 1.01 | 0.44 | | 0.59 | 0.83 | | 0.20 |
| Eating disorder | 0.12 | | 0.01 | 0.11 | 0.04 | | 0.08 | 0.07 | | 0.04 |
| Other disorder | 1.41 | | 0.10 | 1.32 | 0.33 | | 1.08 | 1.14 | | 0.28 |
| **DSM-IV Axis 1 disorder (total)** | **12.63** | | **0.57** | **12.05** | **3.04** | | **9.59** | **9.89** | | **2.74** |
| **Total** | **18.79** | | **0.72** | **18.07** | **4.03** | | **14.76** | **14.90** | | **3.90** |

Notes: other disorder is a sexual or gender identity disorder, dissociative disorder, sleeping disorder, impulse control disorder, a disorder as a result of a somatic illness, or disorder not further specified.
