# Supplementary material for: Potential for substitution of mental health care towards family practices: an observational study
Source: BMC Fam Pract. 2017 Jan 31;18:10. doi: 10.1186/s12875-017-0586-4 (PMC5282718; doi:10.1186/s12875-017-0586-4)
Supplement: Additional file 3: — Table S3. Number of patients treated in secondary care per 1,000 Dutch citizens in 2012. Contains a table with the number of patients with mental health problems treated in 2012 in specialized care, according to diagnosis. (DOCX 16 kb) [file 12875_2017_586_MOESM3_ESM.docx]

Additional file 3: Table S3. Number of patients treated in secondary care per 1,000 Dutch citizens in 2012

|  | **Total** | Axis 2 | | | Axis 3 | | | Axis 4 | | |
| --- | --- | --- | --- | --- | --- | --- | --- | --- | --- | --- |
| Axis 1 |  | | Comorbidity | No comorbidity | | Comorbidity | No comorbidity | | Comorbidity | No comorbidity |
| No diagnosis | 4.00 | | 0.03 | 3.97 | | 0.09 | 3.92 | | 0.26 | 3.74 |
| Adjustment problems | 1.35 | | 0.21 | 1.14 | | 0.44 | 0.91 | | 1.24 | 0.11 |
| Other worries or problems | 3.73 | | 0.45 | 3.28 | | 0.73 | 3.00 | | 3.49 | 0.24 |
| **No DSM-IV Axis 1 disorder (total)** | **9.08** | | **0.69** | **8.39** | | **1.26** | **7.82** | | **4.98** | **4.10** |
| Depressive disorder | 7.81 | | 1.80 | 6.00 | | 2.97 | 4.83 | | 7.22 | 0.59 |
| Bipolar disorder | 1.73 | | 0.36 | 1.37 | | 0.61 | 1.13 | | 1.33 | 0.40 |
| Anxiety disorder | 5.55 | | 1.19 | 4.36 | | 1.74 | 3.81 | | 4.88 | 0.67 |
| Psychotic disorder including schizophrenia | 3.34 | | 0.49 | 2.85 | | 1.06 | 2.28 | | 2.95 | 0.39 |
| Substance or alcohol related disorders | 3.30 | | 0.66 | 2.64 | | 1.01 | 2.29 | | 3.21 | 0.09 |
| Dementia | 1.65 | | 0.09 | 1.56 | | 1.36 | 0.28 | | 1.42 | 0.22 |
| Somatoform disorder | 1.13 | | 0.17 | 0.96 | | 0.48 | 0.65 | | 0.96 | 0.17 |
| Eating disorder | 0.60 | | 0.09 | 0.51 | | 0.35 | 0.25 | | 0.48 | 0.11 |
| Developmental or children disorder | 8.98 | | 0.35 | 8.63 | | 1.26 | 7.72 | | 7.99 | 0.98 |
| Other disorder | 1.07 | | 0.23 | 0.84 | | 0.38 | 0.69 | | 0.95 | 0.12 |
| **DSM-IV Axis 1 disorder (total)** | **34.59** | | **5.44** | **29.15** | | **11.21** | **23.37** | | **31.40** | **3.19** |
| **Total** | **43.67** | | **6.13** | **37.54** | | **12.47** | **31.20** | | **36.38** | **7.28** |

Notes: other disorder is a dissociative disorder, simulated disorder, sexual or gender identity disorder, sleeping disorder, impulse control disorder, or a disorder caused by a somatic illness. Adjustment problems (included in the DSM-IV), often triggered by psychosocial circumstances or not complex, do not need specialist treatment according to the Dutch government. Treatment for adjustment problems is no longer covered by the basic insurance in the Netherlands since 2013. Therefore, we categorized patients with adjustment problems within the group of patients with no psychiatric disorders, who should receive treatment within family practice.
