# Supplementary material for: Potential for substitution of mental health care towards family practices: an observational study
Source: BMC Fam Pract. 2017 Jan 31;18:10. doi: 10.1186/s12875-017-0586-4 (PMC5282718; doi:10.1186/s12875-017-0586-4)
Supplement: Additional file 4: — Table S4. Number of patients referred by FPs and PCPs per1,000 Dutch citizens in 2012. Contains a table with the number of patients with mental health problems referred in 2012 by family physicians and by primary care psychologists to primary and specialized care. (DOCX 16 kb) [file 12875_2017_586_MOESM4_ESM.docx]

Additional file 4: Table S4. Number of patients referred by FPs and PCPs per1,000 Dutch citizens in 2012

|  |  | By FPs | By PCPs |
| --- | --- | --- | --- |
| To primary care | No psychiatric disorder | 7.10 | 0.17 |
|  | Psychiatric disorder | 3.58 | 0.32 |
|  | **Total** | **10.68** | **0.49** |
| To secondary care | No psychiatric disorder | 3.08 | 0.32 |
|  | Psychiatric disorder | 2.34 | 1.38 |
|  | **Total** | **5.41** | **1.71** |
| Total | No psychiatric disorder | 10.18 | 0.49 |
|  | Psychiatric disorder | 5.92 | 1.70 |
|  | **Total** | **16.10** | **2.20** |

Notes: FPs = family physicians. PCPs = primary care psychologists.

Primary care: (other) FP, (other) PCP, or social work.
